# The endophyte *Stenotrophomonas maltophilia* EPS modulates endogenous antioxidant defense in safflower (*Carthamus tinctorius* L.) under cadmium stress

**DOI:** 10.1007/s00203-022-03049-8

**Published:** 2022-06-27

**Authors:** Noura Sh. A. Hagaggi, Usama M. Abdul-Raouf

**Affiliations:** Botany Department, Faculty of Science, Aswan University, Aswan, 81528 Egypt

**Keywords:** Antioxidant, Cadmium, Safflower, *Stenotrophomonas maltophilia*

## Abstract

Cadmium (Cd) pollution in agricultural soils induces oxidative stress in plants that in turn is the foremost limiting factor for agricultural productivity. In past few decades, plant–metal–microbe interaction is of great interest as an emerging environmentally friendly technology that can be exploited to alleviate metal stress in plants. Considering these, in the present study an endophytic bacterium strain EPS has been isolated from the roots of common bean. The present strain was identified as *Stenotrophomonas maltophilia* based on 16S rRNA gene sequence. The strain showed Cd tolerance and Cd-adsorption potentials. The inoculation of strain EPS in safflower seeds significantly enhanced the antioxidant defense of plants under Cd-stress conditions through increasing the levels of antioxidant molecules like phenolics, flavonoids and carotenoids as well as improving the activities of the antioxidative enzymes including guaiacol peroxidase (POX), ascorbate peroxidase (APX) and superoxide dismutase (SOD). The output of this study is that strain EPS inoculation mitigates Cd-induced oxidative stress and consequently it may be beneficial, especially in Cd-contaminated crop fields.

## Introduction

Over wide world, heavy metals accumulation in agricultural soils is a serious problem threats crop production (He et al. 2015; Rizwan et al. 2016). Plants uptake heavy metals from contaminated soils and subsequently transmit along the food chain causing potential threat to animal and human health. (Fryzova et al. 2017). Hyperlevels of heavy metals alter normal plant functions and metabolism causing repression of vital processes such as photosynthesis, respiration, and enzymatic activities (Hossain et al. 2015). On the other hand, high levels of heavy metals can induce excess generation of reactive oxygen species (ROS) as well as cytotoxic compounds, leading to oxidative stress via demolishing the equilibrium between prooxidants and antioxidants within the plant cells (Zengin and Munzuroglu 2005; Hossain et al. 2015; Sytar et al. 2013). This results in cellular damage as well as decreasing plant productivity (Raja et al. 2017).

Cadmium (Cd) is unnecessary element for living organisms, and it is highly toxic to plants and animals even at very low concentrations (Dai et al. 2012). Cadmium mainly originates from industrial processes and phosphate fertilizers, releases into agricultural lands and has long biological half-life (Gill et al. 2013). In plants, the exposure to cadmium induces numerous hazards physiological and growth changes as well as oxidative stress by generating ROS, that react with lipids, proteins, pigments and nucleic acids in the plant cell, leading to cellular damage and consequently decreasing productivity (Romero-Puertas et al. 2004). Furthermore, cadmium also can transfer into human via food chain and can result in kidney, bone and lung diseases (Bernard 2008).

Traditional remediation techniques for heavy metal-contaminated soils are expensive and destructive to environment (Meagher 2000). Therefore, scientists and engineers intensify their efforts to find cost effective and safe technologies (Boyajian and Carreira 1997; Wasay et al. 1998). Most of plant associated microorganisms are metal resistant, whose application in heavy metal-contaminated soils can improve metal immobilization in soils and plant biomass (Ma et al. 2011, 2016). Despite that the applications of some potential bacterial strains to remediate soils contaminated with heavy metals have been reported, it is urgent to search a new microbial resources that can be used efficiently in heavy metals remediation (Tirry et al. 2018).

Safflower (*Carthamus tinctorius* L.) is herbaceous annual plant belongs to family Asteraceae. It is cultivated from prehistoric times throughout many areas with temperate climates over the world including southern Asia, China, India, Iran and Egypt (Dordas and Sioulas 2008; Weiss 2000). Safflower is commercially used for vegetable oil extraction, as well as in the traditional medicine for the treatment of rheumatism, paralysis, vitiligo, psoriasis and mouth ulcers (Delshad et al. 2018). Moreover, it has numerous pharmacological activities i.e., antioxidant, analgesic, anti-inflammatory and antidiabetic activities (Asgarpanah and Kazemivash 2013).

It has been reported that safflower plants can accumulate high levels of Cd in their roots and leaves (Shi et al. 2010; Namdjoyan et al. 2011). Although, some scientific data exists on the antioxidant defense mechanisms in response to cadmium stress in safflower cultivars (Namdjoyan et al. 2011), to our knowledge, there is no study dealing with alleviation of Cd- induced oxidative stress in safflower using bacteria. Therefore, the present work was designed to investigate the potentiality of the endophytic bacterium *Stenotrophomonas maltophilia* strain EPS to alleviate Cd-induced oxidative stress in safflower plants.

## Materials and methods

### Isolation and identification of endophytic bacteria

Healthy fresh roots of common bean plants (*Vigna unguiculata* L.) were collected in sterile plastic bags from Aswan University greenhouse. Immediately, samples were surface- sterilized using 70% ethanol (30 s) followed by 5% sodium hypochlorite (3 min) and then washed three times with sterilized distilled water (Vincent 1970). Under aseptic conditions, roots were crushed in sterilized saline solution. Loopful of the obtained suspension was streaked on the surface of tryptic soy agar and nutrient agar plates. Plates were incubated at 37 °C for 72 h for the appearance of colonies.

The ribosomal (16S rRNA) gene of the selected strain was amplified using 27F and 1492R primers (Frank et al. 2008) in Applied Biotechnology lab at Ismailia, Egypt. PCR product was sent to SolGent Co., Ltd., South Korea for sequencing. Then, the similarity of the obtained sequence was evaluated based on BLAST outputs using NCBI reference sequence database. Neighbor-joining phylogenetic tree of the strain was constructed using MEGA X 10.1.7 software (Kumar et al. 2018).

### Cd tolerance by the strain

The maximum tolerable concentration of cadmium by the strain was determined according to the method of Vashishth and Khanna (2015), with slight modification. Briefly, 10 mL of nutrient broth in glass tubes was supplemented with different concentrations of CdCl_2_ i.e., 0 (control), 50, 100, 150, 200, 250 and 300 mg L^−1^. 10 mL of nutrient broth without CdCl_2_ was used as control. Tubes were inoculated with 1 mL of inoculum (10^7^ CFU mL^−1^), and incubated for 48 h at 37 °C and 150 rpm. The optical density (OD) was measured at 600 nm. The highest concentration of cadmium (CdCl_2_) that allowed visible bacterial growth after 48 h of incubation was considered as the maximum tolerable concentration.

### Production of exopolysaccharides (EPS) by the strain

In 250 mL conical flasks, 50 mL of nutrient broth was inoculated with 1 mL of bacterial suspension (10^7^ CFU mL^−1^), and incubated at 37 °C in a rotary shaker at 150 rpm for 48 h. Cultures were centrifuged at 5000 rpm for 15 min. The total content of EPS in the supernatants were estimated using phenol–sulphuric acid method (Dubois et al. 1956).

## Cd- adsorption ability of the strain

The ability of the whole culture of the present strain (cells and supernatant) for adsorping cadmium was evaluated using the method of Du et al. (2016). 100 mL of the whole culture broth contained 50 and 100 mg L^−1^ of CdCl_2_ was shaken at 120 rpm and 37 °C for 24 h. Cells were then removed by centrifugation. Concentration of the residual, non-adsorbed metal ion in the solution was estimated by atomic absorption spectrophotometer (Thermo Scientific™ iCE™ 3000). Experiment was performed in triplicate. The adsorption efficiency (%) was calculated according to the following formula:

$${\text{Adsorption efficiency }}\left( {\text{\% }} \right) = { }\frac{{\left[ {{\text{Cd}}_{{\text{i}}} - {\text{Cd}}_{{\text{e}}} } \right]}}{{{\text{Cd}}_{{\text{i}}} }} \times 100,$$

where Cd_i_ and Cd_e_ are the concentration of initial and equilibrium Cd ion in the solution (mg L^−1^) respectively.

### Seed inoculation and pot experiment

Seeds of safflower (cv. Giza-1) were obtained from Faculty of Agriculture and Natural Resources, Aswan University. Seeds were surface sterilized with 70% ethanol for 3 min, rinsed three times with sterilized distilled water. Seeds thereafter were soaked in a freshly prepared bacterial suspension (10^8^ CFU mL^−1^) for 1 h, and left to dry before sowing. Seeds used for control were soaked in sterilized distilled water.

Seeds were sown in pots containing an autoclaved mixture of clay and sand (1:1 w/w), with maintaining field capacity at 90%. Pots were kept under normal climatic conditions. After 3 weeks of sowing, five homogenous plants in each pot were subjected to three Cd treatments including 0 (control), 50 and 100 mg L^−1^ of CdCl_2_. After 3 weeks of cadmium exposure, healthy expanded leaf samples were collected, frozen and then used for measuring the defensive non-enzymatic and enzymatic antioxidant activities. The experiment was repeated twice.

### Assessments of non-enzymatic antioxidants

#### Total phenolics

The Folin-Ciocalteu assay described by Singleton et al. (1999) was followed to determine the total phenolic compounds in the leaves extracts. Absorbance was read at 700 nm, and the content of total phenolics was expressed as mg gallic acid equivalents per gram of fresh weight using gallic acid as a reference.

#### Total flavonoids

Aluminum chloride method according to Chang et al. (2002) was used for quantifying the total contents of flavonoids of the extracts. The absorbance was recorded at wavelength 510 nm. The concentration of flavonoids was calculated from quercetin calibration curve as mg quercetin equivalents per gram of fresh weight.

#### Total carotenoids

Pigments were extracted from fresh leaves and their contents were estimated as described by Lichtenthaler and Wellburn (1983). One gram of fresh leaves was macerated in 80% acetone, the supernatant was filtered and makeup to 50 mL with the solvent. The total contents of chlorophylls *a* (Chl *a*), chlorophylls *b* (Chl *b*) and carotenoids were measured by reading the absorbance at wavelengths 646, 663 and 440.5 nm respectively. The content of each pigment was calculated in mg per gram of fresh weight using the following equations:

$$\begin{aligned} {\text{Chl }}a \, \left( {{\text{mg g f}}.{\text{wt}}.^{ - 1} } \right) & = \, \left( {12.21 \times A_{663} } \right) \, - \, \left( {2.81 \times A_{646} } \right) \\ {\text{Chl }}b \, \left( {{\text{mg g f}}.{\text{wt}}.^{ - 1} } \right) & = \left( {20.13 \times A_{646} } \right) \, {-} \, \left( {5.03 \times A_{663} } \right) \\ {\text{Carotenoids }}\left( {{\text{mg g f}}.{\text{wt}}.^{ - 1} } \right) & { = }\left( {4.69 \times A_{440.5} } \right) \, - \, 0.268 \times \, \left( {{\text{Chl}}\;a \, + {\text{ Chl}}\;b} \right). \\ \end{aligned}$$

#### Total antioxidant capacity

Total antioxidant capacity of the ethanolic extracts of the leaves was measured per gram of fresh weight as mg ascorbic acid equivalents using ascorbic acid standard curve, according to phosphomolybdnum assay (Prieto et al. 1999).

#### Assessments of enzymatic antioxidants

Antioxidant enzymes were extracted from fresh leaves according to Cavalcanti et al. (2004) with slight modification. One gram of fresh leaves was homogenized using a mortar in 10 mL of extraction buffer containing 0.2 M of potassium phosphate buffer (pH 7.2), 0.1 mM EDTA and 1 mM phenylmethylsulfonyl fluoride as proteinase inhibitor. The homogenate was filtered. The obtained filtrate was used for enzymatic assays.

#### Catalase (CAT) activity

Catalase activity was estimated by the method of Kato and Shimizu (1987). To 3 mL of the reaction mixture containing 50 mM potassium phosphate buffer (pH 7.0) and 20 mM H_2_O_2_, 100 µl of enzymatic extract was added. The decrease in H_2_O_2_ was followed as decline in optical density at 240 nm. Catalase activity was calculated with the extinction coefficient of H_2_O_2_ (40 mM^−1^ cm^−1^), and expressed as 1 μmol of H_2_O_2_ decomposed per minute under assay conditions.

#### Guaiacol peroxidase (POX) activity

The activity of guaiacol peroxidase enzyme was determined following the method of Kim and Yoo (1996). Briefly, the reaction mixture contained 0.2 mL of enzyme extract, 0.8 mL of phosphate buffer (0.2 M, pH 7.2), 1 mL of guaiacol (15 mM) and 1 mL of hydrogen peroxide (3 mM) was incubated for 10 min at 30 °C. Reaction was terminated using 0.5 mL of H_2_SO_4_ (5%), and the absorbance was read at 470 nm. POX activity was calculated using the extinction coefficient of oxidation product (tetraguaiacol), (*ε*470 = 26.6 mM cm^−1^) as follow:

$${\text{U}}/{\text{mL }} = \left[ {{\text{Change in absorbance }}\min^{ - 1} \times {\text{ Reaction mixture volume }}\left( {{\text{mL}}} \right) \, \times {\text{ Dilution factor}}} \right]/\left[ {\varepsilon 470 \times {\text{ Enzyme extract volume }}\left( {{\text{mL}}} \right)} \right]$$

#### Ascorbate peroxidase (APX) activity

Ascorbate peroxidase activity was evaluated according to Senthilkumar et al. (2021). To 0.8 mL of a reaction mixture contained potassium phosphate buffer (50 mM), ascorbic acid (0.5 mM), H_2_O_2_ (1.0 mM) and EDTA (0.1 mM), 0.2 mL of the enzyme extract was added. After 30 s the decrease in absorbance at 290 nm was followed up to 60 s with an interval of 15 s. One unit of enzyme activity was expressed as the amount of enzyme required to oxidize 1 μmoL of ascorbic acid per minute with absorbance coefficient 2.8 mM cm at 290 nm.

#### Superoxide dismutase (SOD) activity

Superoxide dismutase activity was estimated according to Van Rossun et al. (1997). Three mL of reaction mixture contained 50 mM sodium phosphate buffer (pH 7.6), 0.1 mM EDTA, 50 mM sodium carbonate, 50 μM nitroblue tetrazolium (NBT), 10 μM riboflavin, 12 mM l-methionine and 100 μl of crude extract. Tubes contained the same reaction mixture without enzyme extract used as control. The tubes were placed under two 15 W fluorescent lamps for 15 min to start the reaction. The absorbance was recorded at 560 nm. One unit of SOD activity was defined as the amount of enzyme which reduced the absorbance to 50% compared with the control.

#### Estimation of hydrogen peroxide (H_2_O_2_) content in safflower leaves

To evaluate the H_2_O_2_ content of the leaves, the method of Velikova et al. (2000) was followed. One gram of fresh leaves was homogenated in 10 mL trichloroacetic acid (0.1%) using a mortar and pestle, and then centrifuged. To 0.5 mL of the supernatant, 0.5 mL of potassium phosphate buffer (pH 7.0) and 1 mL of 1 M KI were added. The mixture was vortexed, and the absorbance was read at 390 nm. A calibration curve of different concentrations (µmol) of 30% (v/v) H_2_O_2_ was used as standard.

### Statistical analysis

Experimental data were compared by one-way analysis of variance (ANOVA) and Tukey's HSD test using Minitab software (version 18.1). Values were expressed as means ± standard errors (SEs) of three biological replicates obtained from two independent experiments. Different letter alphabets above the graphs indicate significant differences at *p* ≤ 0.05 between inoculated and non-inoculated plants according to Tukey's HSD test, while similar letters indicate no significant result.

## Results

### Bacterial identification

**Fig. 1:**
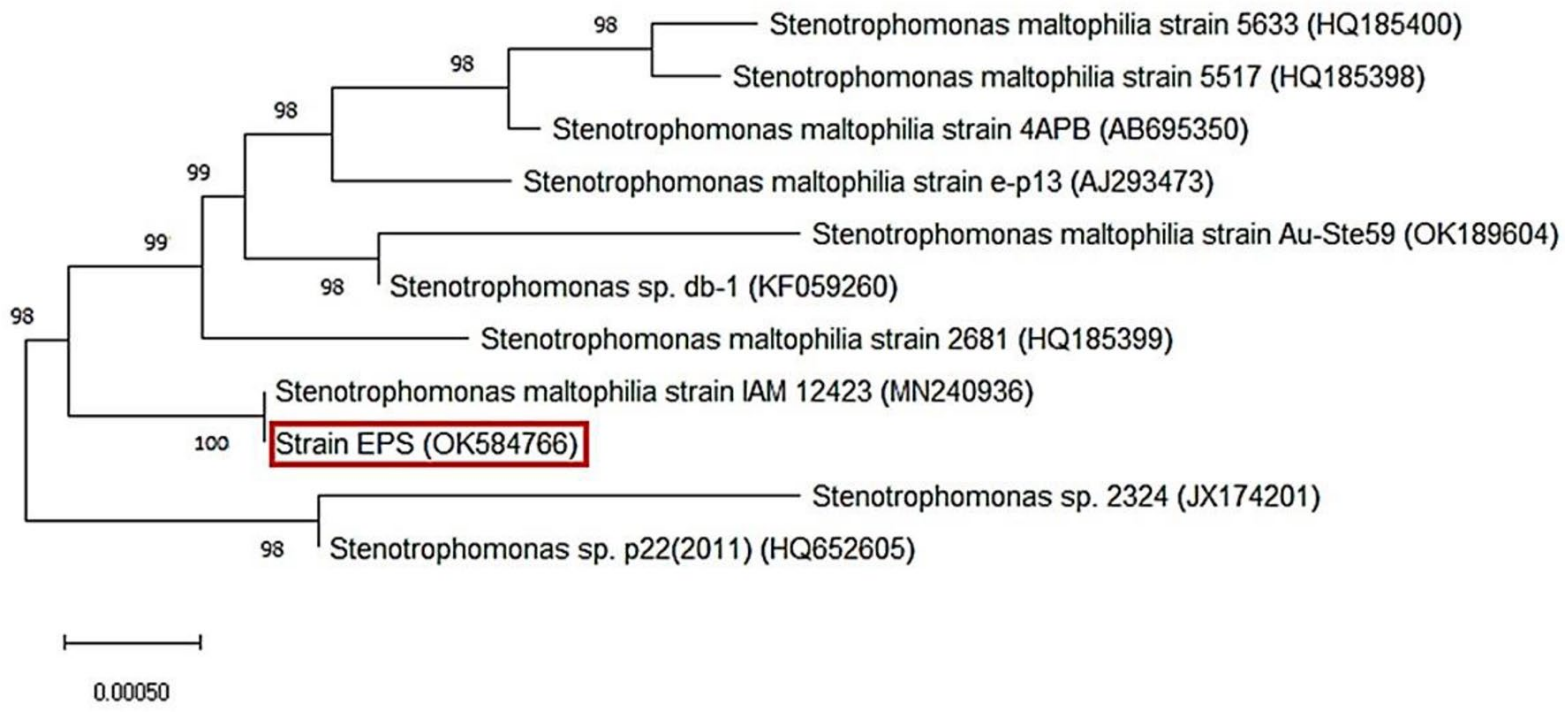
Neighbor-joining phylogenetic tree with 1000 bootstrap replication using MEGA X10.1.7 software displaying the relationship between strain EPS and the closely related members of genus *Stenotrophomonas* derived from NCBI reference sequence database

The selected strain was coded as EPS. The NCBI- BLAST analysis of strain EPS sequence showed closely similarity with percent identity of 100% to *Stenotrophomonas maltophilia* strain IAM 12,423 (MN240936) (Fig. 1). The 16S rRNA gene sequence of strain EPS was deposited to NCBI GenBank with accession number (OK584766).

### Cd tolerance of the strain

**Fig. 2:**
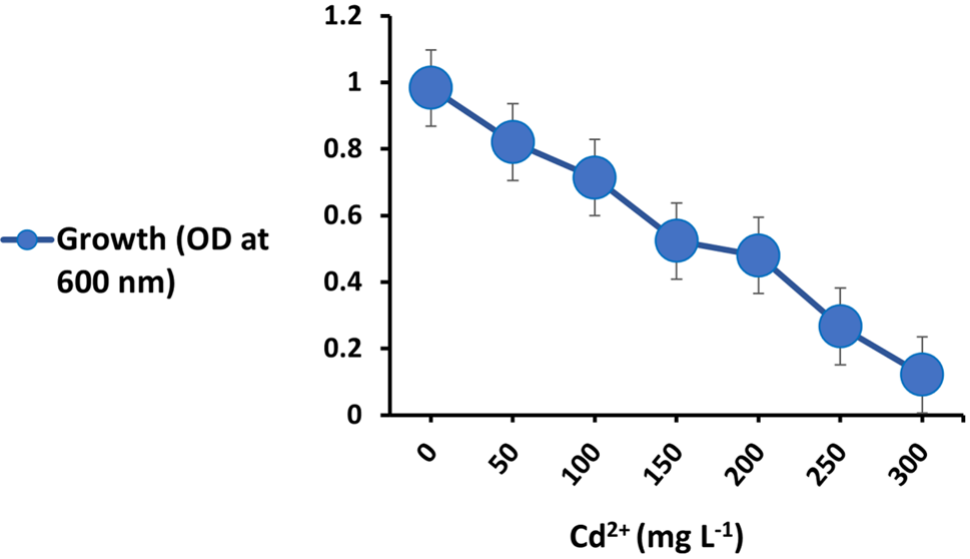
Cd tolerance by strain EPS. Values are means ± SEs of three independent replicates (*n* = 3)

The growth of strain EPS was estimated after 48 h of incubation at different concentrations of CdCl_2_. It was observed that the maximum tolerable concentration was 200 mg L^−1^ CdCl_2_, above this concentration the growth was dramatically declined (Fig. 2).

### Production of exopolysaccharides (EPS) by the strain

Strain EPS exhibited its ability to produce significant amount of EPS. Acorrding to phenol–sulphuric acid assay, the EPS production by the strain was 1.103 ± 0.153 mg glucose equivalent mL^−1^.

### Cd- adsorption ability of the strain

Cd-adsorption efficiency was evaluated using the whole culture of the strain (cells and supernatant) supplemented with 50 and 100 mg L^−1^ CdCl_2_. It was detected that the Cd- adsorption efficiency by the strain was 95.42% and 89.96% in 50 and 100 mg L^−1^ Cd-supplemented culture respectively.

### Effect of bacterial inoculation on antioxidant defense of safflower under Cd stress

**Fig. 3:**
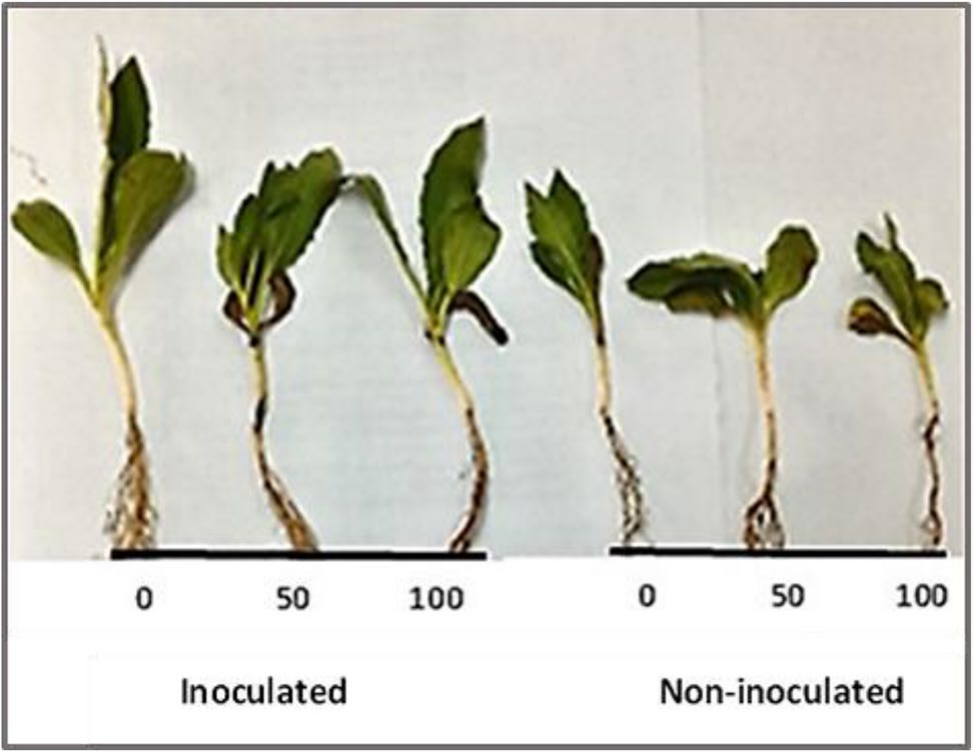
Effect of bacterial inoculation on safflower plants compared to non-inoculated plants at CdCl_2_ concentrations of 0, 50 and 100 mg L^−1^

This study showed that the inoculation of safflower seeds with strain EPS significantly enhanced the antioxidant defense of the plants under Cd stress which directly reflected on plant morphology (Fig. 3).

### Non-enzymatic antioxidants levels

**Fig. 4:**
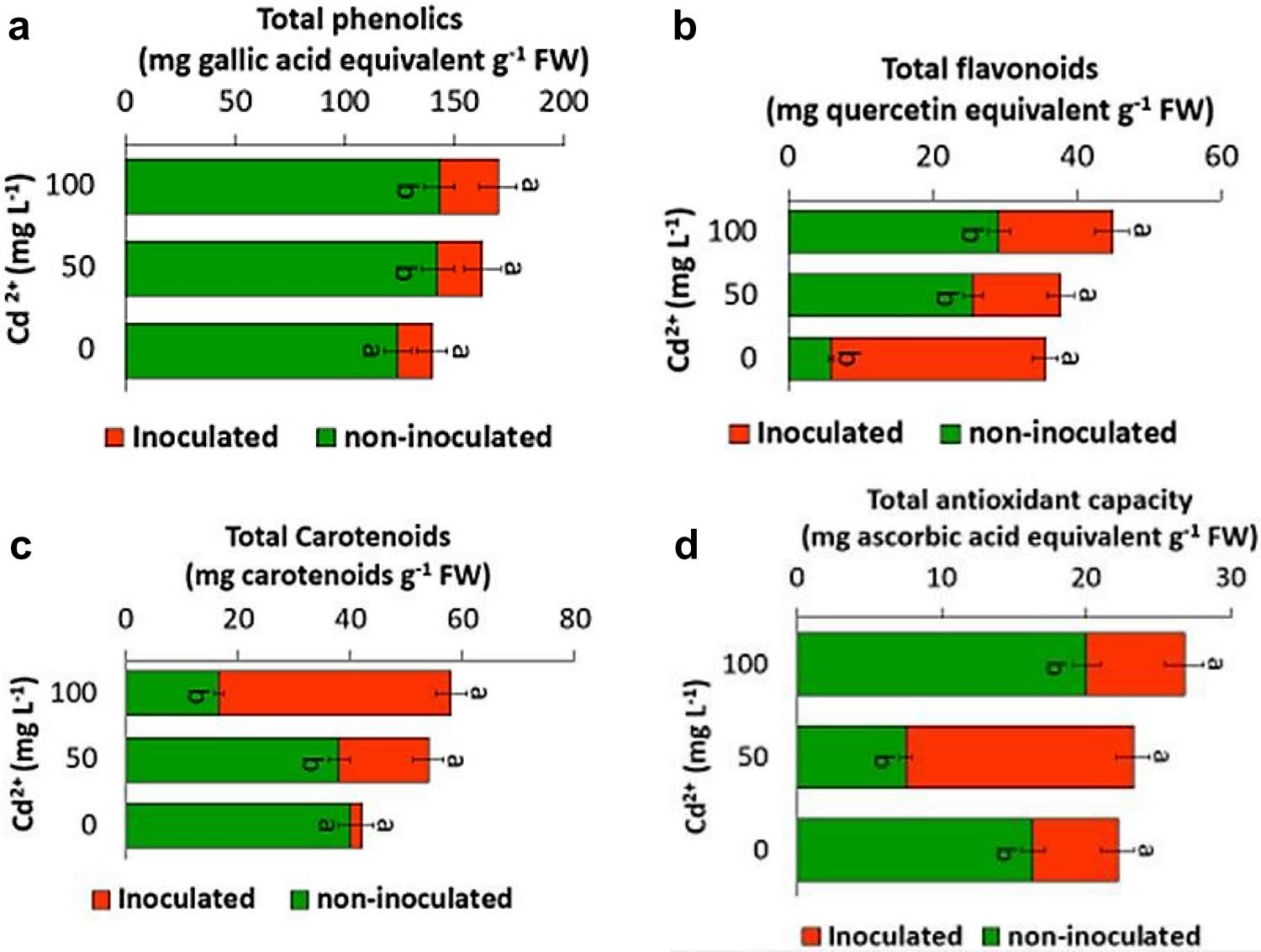
Effect of bacterial inoculation on **a** total phenolics, **b** total flavonoids, **c** total Carotenoids and **d** total antioxidant capacity (mg g^−1^ FW) under Cd stress. Values are means ± SEs of three independent replicates (*n* = 3). Different letter alphabets above the graphs indicate significant differences at *p* ≤ 0.05 between inoculated and non-inoculated plants according to Tukey's HSD test, while similar letters indicate no significant result

In the present study, the total phenolics significantly increased (*f* = 9.11; *p* = 0.0129) with increasing Cd concentration in inoculated plants comparing to non-inoculated plants (Fig. 4a). On the other hand, the inoculation with strain EPS enhanced the total flavonoids content at all the tested Cd concentrations (Fig. 4b). It was found that the content of total flavonoids was increased in inoculated plants by 38.9 and 49.4% over the non-inoculated plants at Cd concentrations of 50 and 100 mg L^−1^ respectively.

Although the content of carotenoids of non-inoculated plants under Cd treatments was remarkably decreased (Fig. 4c), the carotenoids content in inoculated plants was significantly increased (*f* = 12.375; *p* = 0.0055) at all tested Cd treatments (Fig. 4c). Moreover, it was found that the total antioxidant capacity of the inoculated plants was increased by 78.1 and 34% over the non-inoculated plants at 50 and 100 mg L^−1^ CdCl_2_ respectively (Fig. 4d).

### Enzymatic antioxidants levels

**Fig. 5:**
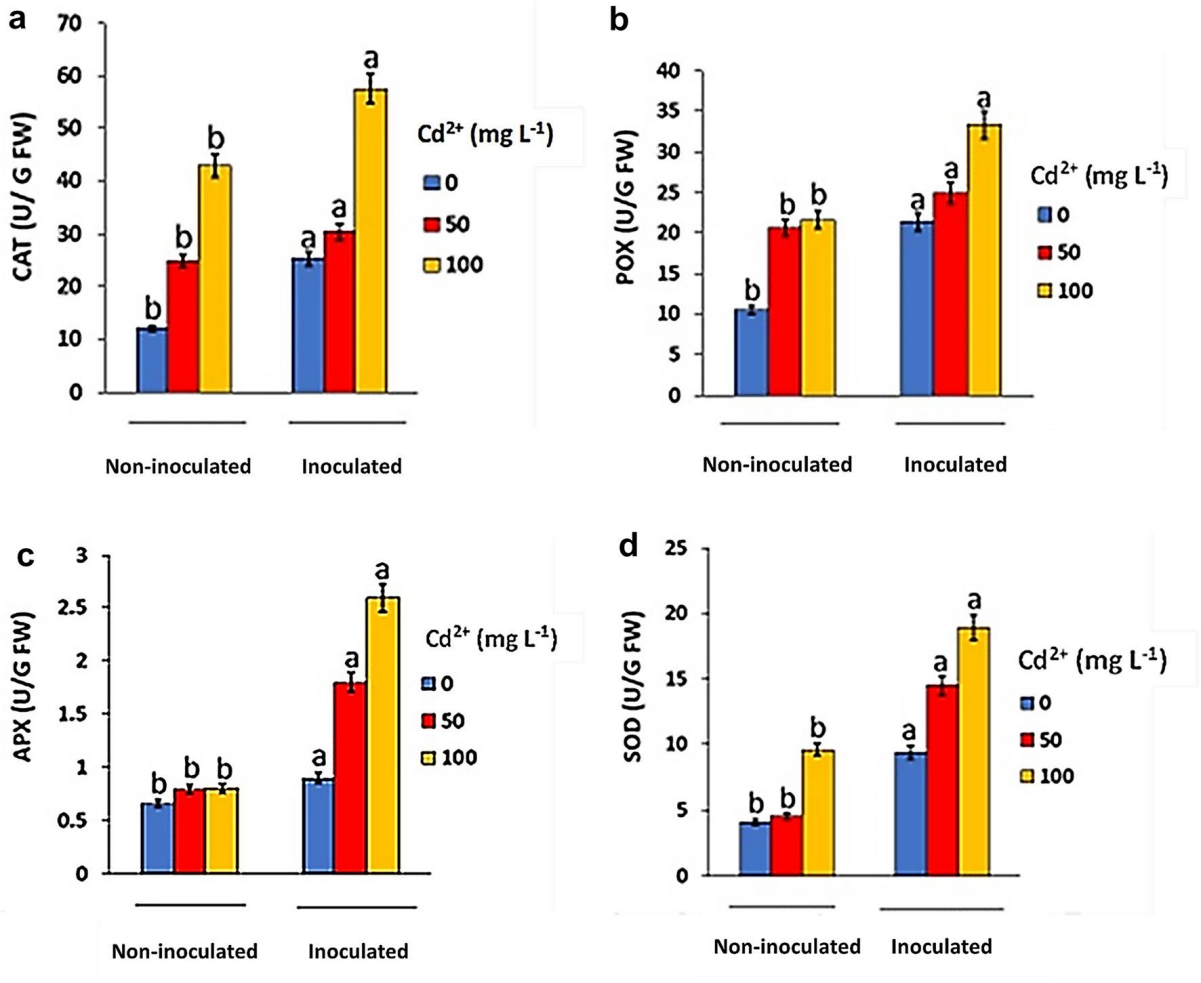
Effect of bacterial inoculation on antioxidant enzyme activities: **a** catalase (CAT), **b** guaiacol peroxidase (POX), **c** ascorbate peroxidase (APX) and **d** superoxide dismutase (SOD). Values are means ± SEs of three independent replicates (*n* = 3). Different letter alphabets above the graphs indicate significant differences at *p* ≤ 0.05 between inoculated and non-inoculated plants according to Tukey's HSD test, while similar letters indicate no significant result

In the current study, the inoculation of safflower with strain EPS significantly (*p* < 0.05) enhanced the activities of CAT, POX, APX and SOD at all the tested Cd levels (Fig. 5a–d). The inoculation enhanced CAT activity in safflower plants by 15–35%. POX activity upon strain EPS inoculation was found to be increased by 20.6–29.6% under Cd stress compared with the non-inoculated plants. The activities of APX and SOD in safflower plants were improved due to the inoculation by 40.5 to 109.9% and 96.9 to 124.6% over the non-inoculated plants under Cd stress, respectively.

### Hydrogen peroxide (H_2_O_2_) content in safflower leaves

**Fig. 6:**
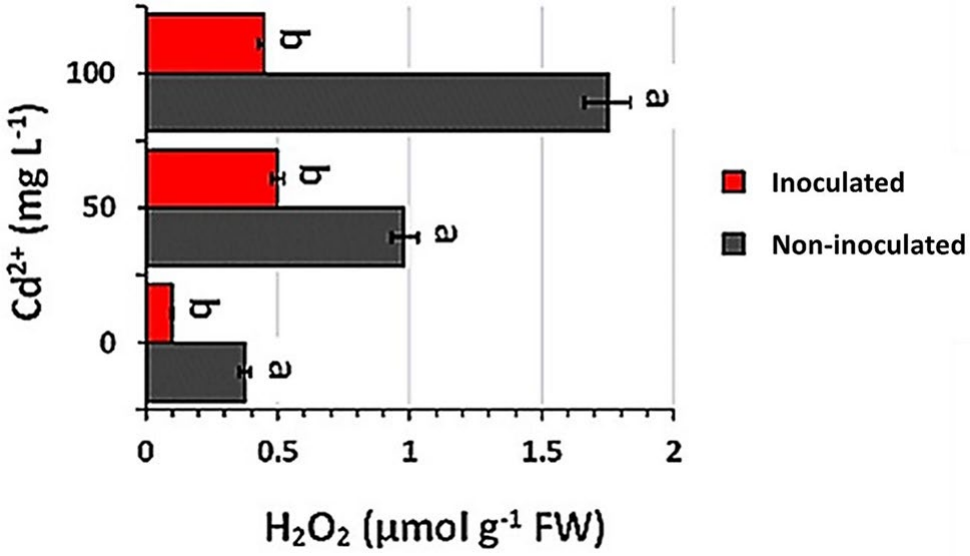
Effect of bacterial inoculation on hydrogen peroxide (H_2_O_2_) content of the leaves under Cd stress. Values are means ± SEs of three independent replicates (*n* = 3). Different letter alphabets above the graphs indicate significant differences at *p* ≤ 0.05 between inoculated and non-inoculated plants according to Tukey's HSD test, while similar letters indicate no significant result

In the current work, it was found that the inoculation of safflower with strain EPS significantly reduced the accumulation of H_2_O_2_ in their leaves under all tested Cd concentrations compared with the non-inoculated plants (Fig. 6).

## Discussion

Metal toxicity and stress in plants triggering the excessive accumulation of ROS in mitochondria, chloroplast, and peroxisomes (Kochian et al. 2004), resulting in imbalance between the generation of ROS and antioxidant defense systems, that in turn causes oxidative stress to plants (Gupta et al. 2013). Oxidative stress disturbs physiological and metabolic processes of the plants leading to a limitation in plant growth, crop production and yield, and consequently causes massive agricultural loss (Tran and Popova 2013). Recently, plant root-associated bacteria are globally used for the amelioration of crop performance to encounter heavy metal contamination in agricultural soils (Mitra et al. 2018; He et al. 2020; Ghosh et al. 2022).

Interestingly, the present strain EPS tolerated Cd up to 200 mg L^−1^ (Fig. 2). Bacteria can tolerate Cd and resist its negative effects using several mechanisms such as transport, precipitation, transformation or intracellular sequestration by thiol containing compounds like metallothionein and glutathione (Intorne et al. 2012; Maynaud et al. 2014). In the present study, strain EPS showed Cd- adsorption ability at both tested Cd concentrations. The ability of strain EPS to adsorb Cd ions may be attributed to the negatively charged functional groups (carboxyl, phosphoryl and hydroxyl) in its polysaccharide structure that can bind the positively charged metal ions. Our finding is in agreement with Liaquat et al. (2020) who reported that *Stenotrophomonas maltophilia* has remarkable Cd- adsorption potential under varying concentrations.

Levels of antioxidants within the plant cell tend to fluctuate at cadmium exposure (Ali et al. 2019). The interaction between plants and microorganisms at biochemical, physiological and molecular levels largely directs plant responses toward abiotic stresses (Farrar et al. 2014; Meena et al. 2017). This crucial aspect considered as an interest gateway for scientists to search novel cost effective and eco-friendly methods to alleviate the abiotic stresses in field grown plants. The application of bacteria to mitigate stress-induced negative impact in plants and their role to make plants tougher toward abiotic stresses have been documented (Panlada et al. 2013; Nadeem et al. 2014; Kaushal and Wani 2016; Rizvi and Khan 2018; Ghosh et al. 2022). In this study, the efect of *S. maltophilia* EPS inoculation on the antioxidant defense of safflower plants (*Carthamus tinctorius* L.) exposed to different levels of Cd was investigated.

The non-enzymatic antioxidants like phenolic compounds, flavonoids, ascorbate as well as carotenoids considered as the half of the antioxidant machinery of the plant cell (Das and Roychoudhury 2014). They play a vital role in the plant cell through protecting the cell components from oxidative damage as well as improving plant growth and development via modifying cellular processes such as mitosis, cell elongation, senescence and cell death (de Pinto and De Gara 2004). Phenolics are better and more efficient antioxidant due to the presence of hydroxyl ions in their structure that can chelate metal ions, trap active oxygen species as well as inhibit lipid peroxidation (Michalak 2006; Ali et al. 2019). Flavonoids are secondary antioxidants with variable phenolic structures that act as reactive oxygen species (ROS) scavengers (Fini et al. 2011; Das and Roychoudhury 2014). In the current study, the inoculation with strain EPS significantly enhanced the total phenolics and total flavonoids contents in safflower plants at all the tested Cd concentrations (Fig. 4a, b).

Carotenoids are lipophilic antioxidants in the plant plastids. They prevent oxidative damage and protect photosynthetic apparatus via detoxifying multiple forms of ROS (McElroy and Kopsell 2009). The exposure to Cd resulted in a decrease of carotenoids contents of safflower plants that attributes to Cd-induced decrease of the photosynthetic rate (Mobin and Khan 2007; Shi et al. 2010). In the present study, the inoculation with strain EPS was significantly improved the quantities of carotenoids antioxidants in safflower plants under Cd stress compared to the non-inoculated plants (Fig. 4c). Moreover, the total antioxidant capacity of safflower plants increased due to bacterial inoculation (Fig. 4d). This is because the total antioxidant capacity was strongly correlated with total phenolics and total flavonoids contents. The positive correlations between total phenolics, total flavonoids and antioxidant activities were reported by other researchers (Gouveia and Castilho 2011; Contreras-Calderón et al. 2011; Aryal et al. 2019; Santos and MagalhÃes 2020; Butkeviciute et al. 2022).

Plants possess multiple antioxidative enzymes including catalase (CAT), guaiacol peroxidase (POX), Ascorbate peroxidase (APX) and superoxide dismutase (SOD) that alleviate oxidative stress and maintain redox homeostasis through catalyting the transformation of ROS into stable nontoxic molecules (Sáez and Están-Capell 2014). In the current study, the inoculation of safflower with strain EPS resulted in significant improvement of the activities of antioxidative enzymes including CAT, POX, APX and SOD (Fig. 5a–d).

As a result of many stresses, the cellular concentration of superoxide radicals increases, which are subsequently converted to hydrogen peroxide by mitochondrial manganese superoxide dismutase (Huseynova et al. 2015). Hydrogen peroxide is one of the major contributors causing oxidative damage to plant cell, leading to inhibition of plant growth and development, or to death (Hung et al. 2005; Hossain et al. 2015). Interestingly, the inoculation with strain EPS led to remarkable reduction in the H_2_O_2_ contents of safflower leaves (Fig. 6). This may be because the activity of catalase enzyme increases with increasing Cd level (Fig. 5 a), as an antioxidant defense to breakdown toxic H_2_O_2_ into water and divalent oxygen (Cuypers et al. 2010).

## Conclusion

An endophytic bacterium *Stenotrophomonas maltophilia* EPS was isolated from common bean roots. The strain exhibited strong Cd tolerance and adsorption efficiency. The strain was inoculated into safflower seeds to evaluate its effect on plant antioxidant defense under Cd stress. The output of the study is that the inoculation was significantly improved the antioxidant defense of safflower plants under Cd stress through increasing the levels of antioxidant compounds and enhancing the activities of antioxidant enzymes. This study provides an eco-friendly and safety method for alleviating Cd stress in plants, that can guarantee safe agricultural productivity in Cd-contaminated fields.
